# *ADAMTS1*, *MPDZ, MVD*, and *SEZ6:* candidate genes for autosomal recessive nonsyndromic hearing impairment

**DOI:** 10.1038/s41431-021-00913-x

**Published:** 2021-06-16

**Authors:** Thashi Bharadwaj, Isabelle Schrauwen, Sakina Rehman, Khurram Liaqat, Anushree Acharya, Arnaud P. J. Giese, Liz M. Nouel-Saied, Abdul Nasir, Jenna L. Everard, Lana M. Pollock, Shaoyuan Zhu, Michael J. Bamshad, Deborah A. Nickerson, Raja Hussain Ali, Asmat Ullah, Abdul Wali, Ghazanfar Ali, Regie Lyn P. Santos-Cortez, Zubair M. Ahmed, Brian M. McDermott, Muhammad Ansar, Saima Riazuddin, Wasim Ahmad, Suzanne M. Leal

**Affiliations:** 1 Center for Statistical Genetics, Gertrude H. Sergievsky Center, and the Department of Neurology, Columbia University Medical Center, New York, NY USA; 2 Department of Otorhinolaryngology – Head and Neck Surgery, University of Maryland, Baltimore, MD USA; 3 Synthetic Protein Engineering Lab (SPEL), Department of Molecular Science and Technology, Ajou University, Suwon, South Korea; 4 Case Western Reserve University, Department of Otolaryngology, Head and Neck Surgery, Cleveland, OH USA; 5 Department of Genome Sciences, University of Washington, Seattle, WA USA; 6 Department of Pediatrics, University of Washington, Seattle, WA USA; 7 Department of Hematology/Oncology, Boston Children’s Hospital, Boston, MA USA; 8 Novo Nordisk Foundation Center for Basic Metabolic Research, Faculty of Health and Medical Sciences, University of Copenhagen, Copenhagen, Denmark; 9 Department of Biotechnology and Informatics, Faculty of Life Sciences and Informatics, BUITEMS, Quetta, Pakistan; 10 Department of Biotechnology, University of Azad Jammu and Kashmir, Muzaffarabad, Pakistan; 11 Department of Otolaryngology – Head and Neck Surgery, School of Medicine, University of Colorado Anschutz Medical Campus, Aurora, CO USA; 12 Department of Biochemistry, Faculty of Biological Sciences, Quaid-i-Azam University, Islamabad, Pakistan; 13 Taub Institute for Alzheimer’s Disease and the Aging Brain, Columbia University Medical Center, New York, NY USA

**Keywords:** Genetics, Genetics research

## Abstract

Hearing impairment (HI) is a common disorder of sensorineural function with a highly heterogeneous genetic background. Although substantial progress has been made in the understanding of the genetic etiology of hereditary HI, many genes implicated in HI remain undiscovered. Via exome and Sanger sequencing of DNA samples obtained from consanguineous Pakistani families that segregate profound prelingual sensorineural HI, we identified rare homozygous missense variants in four genes (*ADAMTS1*, *MPDZ, MVD*, and *SEZ6*) that are likely the underlying cause of HI. Linkage analysis provided statistical evidence that these variants are associated with autosomal recessive nonsyndromic HI. In silico analysis of the mutant proteins encoded by these genes predicted structural, conformational or interaction changes. RNAseq data analysis revealed expression of these genes in the sensory epithelium of the mouse inner ear during embryonic, postnatal, and adult stages. Immunohistochemistry of the mouse cochlear tissue, further confirmed the expression of ADAMTS1, SEZ6, and MPDZ in the neurosensory hair cells of the organ of Corti, while MVD expression was more prominent in the spiral ganglion cells. Overall, supported by in silico mutant protein analysis, animal models, linkage analysis, and spatiotemporal expression profiling in the mouse inner ear, we propose four new candidate genes for HI and expand our understanding of the etiology of HI.

## Introduction

Hearing impairment (HI) is a highly heterogeneous and common sensory disorder [1]. The prevalence of congenital HI is estimated to be 1 in 500 newborns [2]. To date >80 genes have been implicated in autosomal recessive (AR) nonsyndromic HI (NSHI). Close to a third of ARNSHI genes have been identified via the study of large Pakistani consanguineous families [2]. Unraveling the genetic etiology of HI is vital to aid in genetic counseling, developing, and delivering therapeutic interventions. We identified homozygous missense variants in candidate genes: *ADAMTS1* (OMIM: 605174); *MPDZ* (OMIM: 603785); *MVD* (OMIM: 603236); and *SEZ6* (OMIM: 616666), which are likely to be the underlying cause of AR sensorineural profound NSHI in four consanguineous Pakistani families. These candidate genes were identified and studied using a combination of technologies that included Sanger and exome sequencing, linkage analysis, and expression studies.

## Materials and methods

### Sample collection and clinical evaluation

Approval from the Institutional Review Boards of Quaid-i-Azam University (IRB-QAU-153) and Columbia University (IRB-AAAS2343) were obtained for the study. All adult study participants signed informed consent forms and parents provided consents for minors after their assent was obtained. The families were ascertained from Azad Jammu and Kashmir (AJK) (family 4140), Punjab (families 4444 and 4457), and Sindh (4876) provinces. Participant evaluation included obtaining a family and clinical history, available medical records, physical examination, and pure-tone audiometry (250–8000 Hz). Tandem gait and Romberg tests were used to determine if the hearing-impaired family members had gross vestibular dysfunction. Further clinical evaluation was performed to rule out syndromes or HI due to infections, ototoxic medications, or trauma. Peripheral blood samples were collected from all informative participating family members and genomic DNA was extracted [3].

### Exome sequencing, variant confirmation, and linkage analysis

**Fig. 1:**
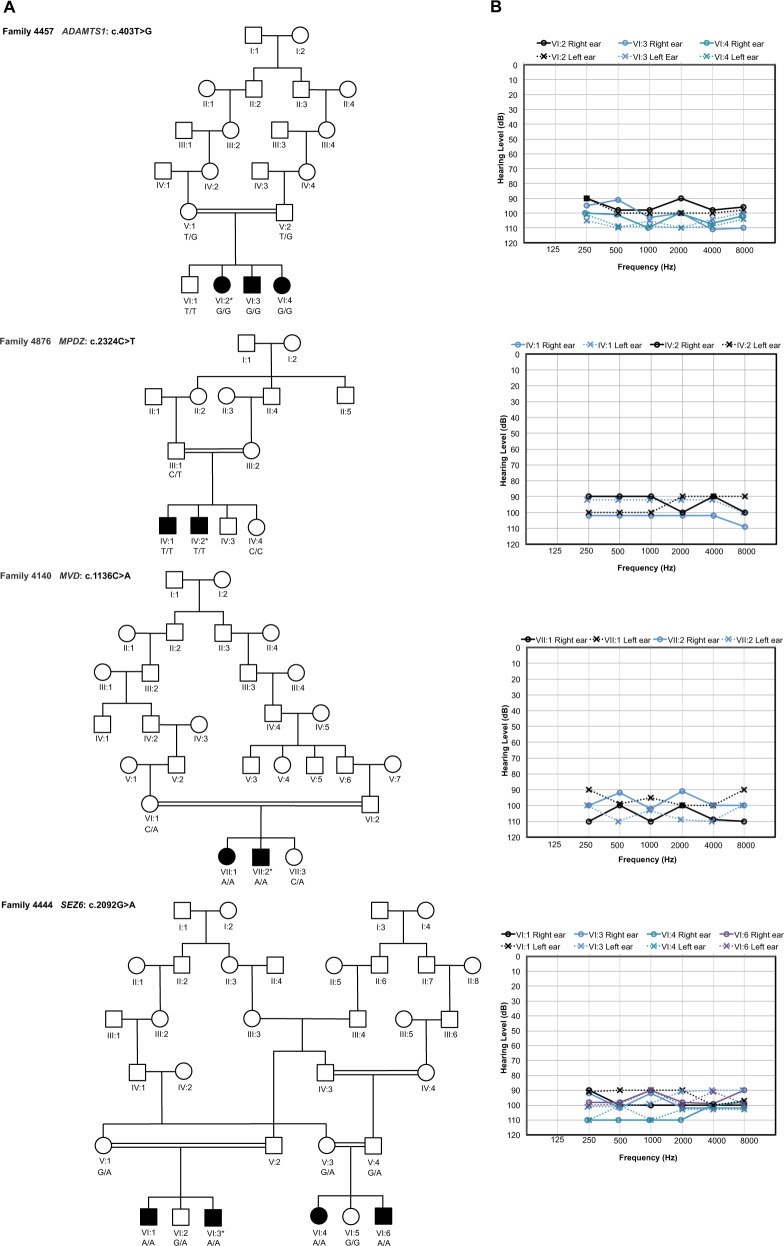
Pedigrees and audiometry for affected family members. **A** Pakistani pedigrees 4457, 4876, 4140, and 4444 displaying genotypes for the candidate genes for each family member that has an available DNA sample. A star indicates the affected family member whose DNA underwent exome sequencing. Females are represented by circles and males by squares, those individuals with solid symbols have NSHI while those with clear symbols are unaffected. **B** Air conduction thresholds for affected members of the four families. All individuals had profound bilateral NSHI. Circles with smooth connecting lines represent the right ear and crosses with dotted connecting lines, the left ear. The audiogram of each affected individual is represented by a different color.

To exclude common genetic causes of HI in the Pakistani population, the coding exons of *GJB2* and variants in *CIB2* [p.(Phe91Ser) and p.(Cys99Trp)], *HGF* (c.482 + 1986_1988delTGA and c.482 + 1991_2000delGATGATGAAA), and *SLC26A4* [p.(Val239Asp) and p.(Gln446Arg)] were Sanger sequenced using DNA samples from family members with HI. Subsequently a DNA sample from an affected family member (4140-VII:2, 4444-VI:3, 4457-VI:2, and 4876-IV:2) from each pedigree was exome sequenced (Fig. 1A). Library construction and exon capture were completed using Roche/Nimblegen SeqCap EZ v3.0 kit (~64 Mb target) (4140-VII:2 and 4457-VI:2); Roche/Nimblegen SeqCap EZ v2.0 kit (~37 Mb target) (4444-VI:3); and Agilent SureSelect Human All Exon V6 kit (~60 Mb target) (4876-IV:2). Reads were aligned to the human reference genome (GRCh37/hg19) using Burrows-Wheeler Aligner [4]. Duplicate reads were marked using Picard. Single nucleotide variants and insertions/deletion were called using the Genome Analysis Toolkit [5]. Annotation was performed using ANNOVAR [6], dbNSFP35a [7], and dbscSNV1.1 [8]. Rare variants [population specific minor allele frequency (MAF) < 0.005 in the Genome Aggregation Database (gnomAD) [9] that were consistent with an AR mode of inheritance and were predicted to affect protein function or pre-mRNA splicing (nonsense, frameshift, missense, start-loss, splice region, etc.) were validated and tested for segregation. Copy number variant (CNV) calls were generated using CONiFER (v0.2.2) [10] and annotated using the BioMart Database [11]. Filtering was performed using a population specific MAF < 0.005 in Database of Genomic Variants [12] and gnomAD. Variants that met the above criteria were visualized using the Integrative Genomics Viewer [13] to exclude likely false-positive calls. Sanger sequencing, using DNA samples from all family members, was performed to validate variants and test for segregation with the NSHI.

After Sanger sequencing, Superlink-Online SNP 1.1 [14] was used for each family to perform two-point linkage analysis for each identified candidate variant. An AR mode of inheritance with complete penetrance and south Asian gnomAD allele frequencies were used to calculate LOD scores at theta=0 (Table 1).

**Table 1:** Information on the variants found in the four candidate NSHI genes.

Gene	*ADAMTS1*	*MPDZ*	*MVD*	*SEZ6*
Family	4457	4876	4140	4444
Chromosome	21	9	16	17
Genomic Location (hg 19 position)	g.28216871	g.13188823	g.88719000	g.27286058
Reference Allele	A	G	G	C
Alternate Allele	C	A	T	T
cDNA Change	c.403T>G	c.2324C>T	c.1136C>A	c.2092G>A
Transcript Number	NM_006988.5	NM_003829.4	NM_002461.3	NM_178860.5
Protein Change	p.(Ser135Ala)	p.(Pro775Leu)	p.(Pro379His)	p.(Val698Ile)
gnomAD Frequency ALL	1.00 × 10^–4^	2.81 × 10^–5^	5.97 × 10^–6^	1.00 × 10^–4^
gnomAD Frequency SAS	9.00 × 10^–4^	0	4.28 × 10^–5^	9.00 × 10^–4^
CADD Score	25.20	19.87	25.10	24.50
GERP Score	4.16	5.01	4.56	4.71
Bioinformatic tools with damaging results	MT, PR, SI, PP2, M-CAP, Fathmm-MKL	MT, LRT, PR, SI, Fathmm-MKL	MT, LRT, PR, SI, PP2, mSVM, M-CAP, Fathmm-MKL	MT, LRT, Fathmm-MKL
LOD Score	3.56	1.93	3.70	4.97

### In silico protein analysis and 3D modeling

Structural and functional influence of the identified variants were assessed through a combination of the following in silico tools, including but not limited to: prediction scores from dbnsfp35a, I-MUTANT 3.0 server, PROVEAN-Protein Variation Effect Analyzer, PhD-SNP-Predictor of human Deleterious Single Nucleotide Polymorphisms, SNPs&GO, and Mutation Taster. To predict the effect of variants on the 3D structure of ADAMTS1, MPDZ, MVD, and SEZ6 proteins, homology models were constructed for both native and mutant types using SWISS-MODEL and I-TASSER server [15]. Crystallographic structures corresponding to the following Protein Data Bank [16] IDs; 6O38, 2D92, 3D4J, and 5O32 were used as template for modeling ADAMTS1, MPDZ, MVD, and SEZ6 protein structures, respectively. The structures were visualized and prepared using the PyMOL program.

### Immunohistochemistry of the mouse inner ear

Inner ear tissues were dissected from wildtype mice at P1, P4, P12, and P28 stages. The tissues were fixed in 4% paraformaldehyde in phosphate buffered saline (PBS) overnight at 4 °C and subsequently decalcified in 0.25 M EDTA solution overnight at 4 °C. For whole mount immunostaining, the cochlea was micro-dissected. Dissected tissues were embedded in an optimal cutting temperature medium and sectioned on a cryostat to 5 μm sections. Next, specimens were blocked with 10% normal serum in PBS containing 0.25% Triton X-100 (1 h at room temperature), followed by overnight incubation at 4 °C with a primary antibody (1:200) in 3% normal serum in PBS. After incubation with a primary antibody, specimens were washed with 0.25% Triton X-100 in PBS for three times and incubated with the secondary antibody for one hour at room temperature. The wash step was repeated after the incubation with secondary antibodies. F-actin labeling was done using phalloidin 488 nm and rhodamine phalloidin (1:300 dilution) (Invitrogen, Thermofisher Scientific, Waltham, MA, USA). Confocal images were acquired from a Nikon spinning disk W1 confocal microscope and images were processed using ImageJ software. The following primary antibodies were used: sheep anti-mouse ADAMTS1 (AF5867-SP; Novus Biologicals, Centennial, CO, USA), rabbit anti-mouse MPDZ (42-2700; Thermofisher Scientific, Waltham, MA, USA), rabbit anti-mouse MVD (PA5-22164; Thermofisher Scientific), and sheep anti-mouse SEZ6 (PA5-47683; Thermofisher Scientific). The following secondary antibodies were used: Donkey anti-sheep 564 nm for ADAMTS1 and SEZ6 (Invitrogen), Goat anti-rabbit 594 nm for MPDZ (Invitrogen), and Goat anti-rabbit 488 nm for MVD (Invitrogen).

### RNA expression profiling of mouse inner ear tissues

To evaluate the RNA expression of *Adamst1, Mpdz, Mvd*, and *Sez6* in the mouse inner ear, publicly available datasets in Gene Expression Omnibus (GEO) database, SHIELD (Shared Harvard Inner-Ear Laboratory Database), and gEAR (gene Expression Analysis Resource) websites were studied in silico. First, RNA-sequencing data of hair cells and surrounding cells in the mouse cochlea and utricle at four developmental stages (E16, P0, P4, and P7) were analyzed [17]. Next, a cell specific transcriptome analysis dataset (GSE111347) that includes the transcriptome of adult pillar and Deiters’ cells of adult CBA/J mice measured via RNA-sequencing was examined [18]. Single cell expression data obtained from the cochlear floor epithelial duct cells of E14, E16, P1, and P7 wild type CD-1 mice pups (accession number GSE137299) were analyzed as well [19]. The expression profile across inner ear epithelial cells for each gene was created through the gEAR analysis suite. We also analyzed the expression of the NSHI candidate genes in adult CBA/J mice (25–35 days after birth) cochlear inner hair cells (IHC) and outer hair cells (OHC), using processed expression data from the study GSE56866 [20] in the GEO database. Lastly, a microarray dataset published by Lu et al. [21] containing the spiral and vestibular ganglion neurons collected at six developmental stages (E12, E13, E16, P0, P6, and P15) were also examined. The dataset obtained from SHIELD contained RNA microarrays of E12 and E13 spiral and vestibular ganglia, as well as E16, P0, P6, and P15 vestibular ganglia from mice embryos or pups.

## Results

### Clinical evaluation

The clinical examinations and pure-tone audiometry of the affected members of the four families (Fig. 1A) display profound bilateral HI across all frequencies in both ears (Fig. 1B). The onset of HI was prelingual and most likely congenital. There were no indications that the HI is part of a syndrome.

### Exome sequencing and Sanger sequencing

Of the rare ARNSHI variants that were identified (Supplementary Table 1) through exome sequencing only four homozygous variants in *ADAMTS1* (NM_006988.5) [c.403T>G:p.(Ser135Ala)] family 4457; *MPDZ* (NM_003829.4) [c.2324C>T:p.(Pro775Leu)] family 4876; *MVD* (NM_002461.3) [c.1136C>A:p.(Pro379His)] family 4140; and *SEZ6* (NM_178860.5) [c.2092G>A:p.(Val698Ile)] family 4444 segregated with HI (Fig. 1A, Table 1 and Supplementary Table 2). None of the other identified variants segregated with NSHI in these families. We also did not identify CNVs that are homozygous or in compound heterozygosity with potentially damaging heterozygous SNVs or indels in the same gene. A significant LOD score (>3.3) was obtained for each variant at Θ=0 (LOD = 3.56 [c.403T>G:p.(Ser135Ala), *ADAMTS1*]; LOD = 3.70 [c.1136C>A:p.(Pro379His), *MVD*]; and LOD = 4.97 [c.2092G>A:p.(Val698Ile), *SEZ6*]) with the exception of the c.2324C>T:p.(Pro775Leu) *MPDZ* variant for which only a suggestive LOD score of 1.93 was observed (Table 1).

### In silico protein analysis and 3D modeling

**Fig. 2:**
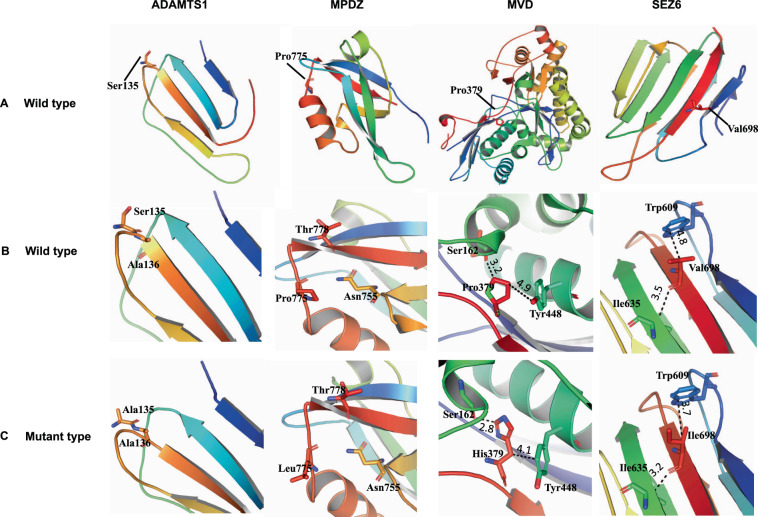
Structural modeling of wild type and mutant proteins. Panel **A**: Structural model of the wild type ADAMTS1, MPDZ, MVD, and SEZ6 proteins. Panel **B**: Interacting bond of the wild type proteins for the four candidate genes. Panel **C**: Mutant type proteins highlighting the change due to the variant. The variants ADAMTS1 [p.(Ser135Ala)] and MPDZ [p.(Pro775Leu)] may result in a difference in interaction pattern with the solvent molecules. As the position of the variant residue is at the loop region, they do not involve a direct interaction, but the native fold of the protein may be affected. MVD [p.Pro379His] mutated protein shows a difference in interacting bond distance in the mutant type from the wild type and may perturb the amino acid side chain. The SEZ6 [p.Val698Ile] mutant protein displayed less interaction distance due to substitution of Val to Ile at position 698, while the SEZ6 wild type protein has a weak H-bond and hydrophobic interaction with Ile635 and Trp609. This variation could possibly change the orientation of the amino acid residue. Structures are displayed as ribbon while residue is represented by stick model. The structure illustrations were created with the PyMOL program. Black dotted lines represent hydrogen bonds.

Homology modeling and protein threading techniques were utilized to predict the wild and mutant type three-dimensional protein structures of ADAMTS1, MPDZ, MVD, and SEZ6 (Fig. 2). The p.Ser135 of ADAMTS1 resides on the surface loop region and the polar side chain replaced by the small non-reactive side chain of alanine in ADAMTS1 [p.(Ser135Ala)] might change the solvent accessible surface area of the mutant protein. For wild-type MDPZ Proline is commonly found in turns, hence its observed replacement by Leucine might yield considerably destabilizing conformational changes to the native MDPZ folding. For MVD a substitution of a proline with histidine at amino acid position 379 was observed. The p.Pro379 is one of the nonpolar aliphatic amino acids, reside at β- strand while histidine has an imidazole aromatic ring, an ionizable side chain, which allows histidine to have a variety of interactions such as cation-π, hydrogen-π and π-π stacking interactions. The substitution by the less hydrophobic and more flexible histidine could induce local structural confirmation as displayed by difference in the interaction distance with the partner interacting residues. In case of [p.(Val698Ile)] for SEZ6, which resides in the β-sheet, a smaller aliphatic side chain replaced by a larger aliphatic side chain may fill the space in the protein core. The difference in interaction distance may cause slight destabilization of the SEZ6 protein but not totally damage the protein structure.

### Expression of candidate genes in the mouse inner ear

**Fig. 3:**
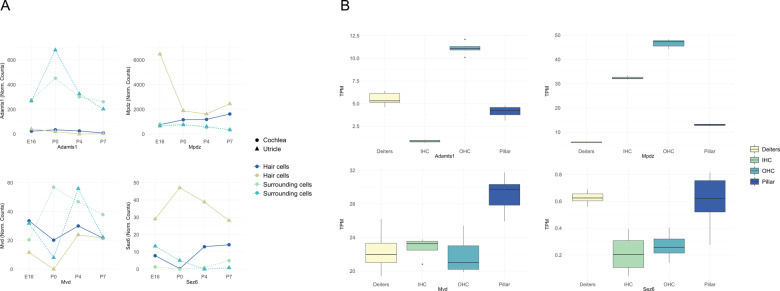
RNA expression of the four candidate genes in the mouse inner ear cells at developing and adult stages. **A** RNA expression of the four candidate genes in the cochlea and utricle during mouse development. RNA-sequencing data of hair cells (GFP+) and surrounding cells (GFP−) from the cochleae and utricles of mice expressing EGFP under the Pou4f3 promoter. Data were collected at four developmental stages: E16, P0, P4, and P7 and RNA expression is represented as normalized counts. *Adamts1*, *Mpdz*, *Mvd*, and *Sez6* were expressed in the hair cells and surrounding cells of the cochlea and utricle during the E16, P0, P4, and P7 stages. **B** RNA expression of the four candidate genes in the adult inner ear cells. The figure represents expression of the genes of interest in Deiters’ cells (Deiters), Pillar cells (Pillar), Inner Hair Cells (IHC), and Outer Hair Cells (OHC) of adult CBA/J mice. The four candidate genes *Adamts1, Mpdz, Mvd*, and *Sez6* were expressed in the IHC, OHCs, and Deiter’s and Pillar cells. The y-axis represents the gene expression normalized to transcripts per million (TPM). The dataset was obtained from the GEO database (accession number GSE111347).

**Fig. 4:**
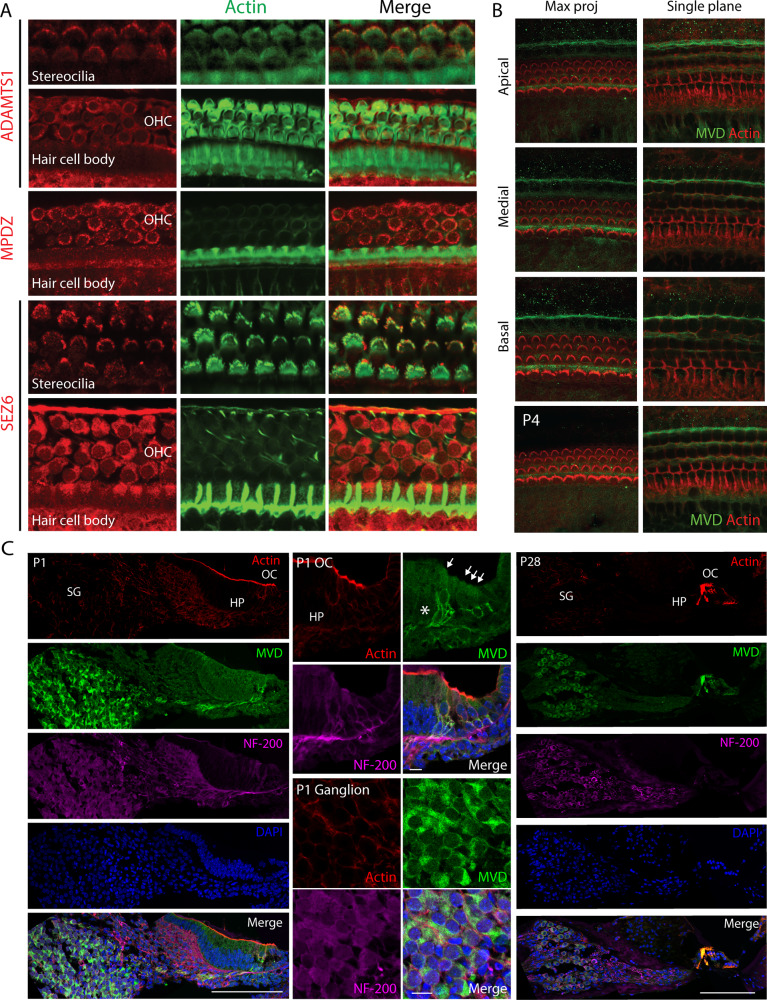
Immunostaining of Adamts1, Mpdz, Mvd, and Sez6 in the inner ear of wildtype mice. **A** Whole mount immunostaining of Adamts1, Mpdz, and Sez6 in wildtype mice at P12. Immunoreactivity was visualized with a fluorescently labeled secondary antibody (red) and F-actin was stained with phalloidin 488 nm (green). Immunolabeling of Adamts1 and Sez6 is observed in stereocilia as well as cytoplasm of outer hair cells. Mpdz is observed in the cytoplasmic region of hair cells. **B** Immunostaining of the organ of Corti at the apical, medial and basal turns of the cochlea at P4. **C** Immunostaining of Mvd in wildtype mice at P1, P4, and P28. Immunoreactivity of Mvd was visualized with a fluorescently labeled secondary antibody (green), F-actin was stained with rhodamine-phalloidin (red) and nuclear bodies were stained with DAPI (blue). The anti-neurofilament (NF-200, purple) was used to mark the neurons. Immunolabeling of Mvd is observed in spiral ganglion cells (SG). HP Habenula perforata, OC Organ of Corti.

The four genes are expressed either ubiquitously, during the stages of development of the ear or in parts of the inner ear that can impact hearing (Table 2). The analysis with the various data sets available in GEO and gEAR databases show that the orthologous genes of *ADAMTS1, MPDZ, MVD*, and *SEZ6* are expressed throughout the cochlear epithelium during the E14, E16, P1, and P7 (Supplementary Figs. 1–5). Additionally, the analysis of hair cell data sets showed that these genes are expressed in IHC during E14-P1 (Supplementary Figs. 6 and 7) and in the OHC during E14-P7 (Supplementary Figs. 8 and 9) stages of CD-1 mice. *Sez6* expression was particularly localized in the IHC (Supplementary Figs. 6 and 7) and OHC (Supplementary Figs. 8 and 9) during E16, P1, and P7 (Supplementary Figs. 2–5) for the CD-1 mice. Localized expression of *Sez6* was also observed in inner phalangeal cells at E16 and outer sulcus cells at P7 (Supplementary Figs. 2 and 4). In the cochlea and utricle (Fig. 3A) the expression of the four genes varied during the developmental stages (E16, P0, P4, and P7). *Adamts1* is expressed in the surrounding cells of the cochlea and utricle. *Mpdz* is mainly expressed in the hair cells of the cochlea over all four developmental stages, with highest expression during E16. *Mvd* shows a variable expression in the surrounding cells of the cochlea and utricle during development. *Sez6* displays the highest expression in the hair cells of the utricle during development (Fig. 3A). The expressions of all four genes were detected in adult IHC, OHC (Supplementary Fig. 10), pillar and Deiters’ cells in adult CBA/J mice (Fig. 3B). The four candidate genes were also expressed in the spiral and vestibular ganglions (Supplementary Fig. 11). Immunohistochemistry at P12 demonstrates that Adamts1 and Sez6 are expressed in stereocilia as well as the cytoplasm of the outer hair cells (Fig. 4A). Mpdz/Mupp1 is expressed in the cytoplasmic region of hair cells at P12 (Fig. 4A). At P1, P4, and P28, Mvd is expressed in the spiral ganglion cells (Fig. 4B and C).

**Table 2:** Functions, expression and animal model phenotypes for the four NSHI candidate genes.

Gene name	Full name	Inner-ear expression (from SHIELD)				Animal model phenotype	Human disease	Gene description	References
		Adult cochlear IHC (mouse)	Otic progenitor cells (mouse)	Auditory/Vestibular ganglion neurons (mouse)					
				Spiral ganglion	Vestibular ganglion				
*ADAMTS1*	A disintegrin and metallopeptidase with thrombospondin type 1 motif 1	✓	✓	✓	✓	Enlarged renal calices; Abnormal adrenal medullary structure; Impaired growth	None	Matrix metalloproteases in apical cochlear sensory epithelium; Upregulated after intense noise exposure; Linked to follicular rupture, cancer cachexia and several inflammatory processes.	[22, 23]
*MPDZ*	Multiple PDZ domain crumbs cell polarity complex component	✓	✓	✓	✓	Increased threshold for auditory brainstem response in homozygous mice at almost all frequencies	Congenital hydrocephalus - with or without brain or eye anomalies	Modulates synaptic plasticity at excitatory synapses; Mild hearing loss at all frequencies in mice; Controls potentiation of a receptor ion coupled channel gating Ca2+ and Na2+ flow into the cell (AMPA receptor); Protein–protein interactions; Part of sertoli-sertoli junction and tight junction pathways	[24–29]
*MVD*	Mevalonate diphosphate decarboxylase	✓	✓	✓	✓	Preweaning lethality	Porokeratosis - rare skin disorder	Necessary for first committed step in isoprenoid biosynthesis; Cholesterol pathway regulation; Essential for metabolic pathways of growth, neural development, reproduction, vision etc.	[30–37]
*SEZ6*	Seizure-related 6 homolog	✓	✓	✓		Irregular, decreased excitatory postsynaptic current in cerebellum; shortened dendrites; motor impairment	Suggested susceptibility factor for febrile seizures	Regulates excitatory postsynaptic membrane potential; Cortical neurogenesis and neuronal maturation in mice; May play role in cell-cell recognition.	[38–42]

## Discussion

We identified candidate HI genes, *ADAMTS1, MPDZ*, *MVD, and SEZ6*, via exome sequencing of DNA samples obtained from four consanguineous Pakistani families that segregate ARNSHI. The affected family members exhibit profound HI with no additional phenotypic features. To further support the role of these genes in hearing we evaluated the gene expression in the mouse inner ear via immunohistochemistry and RNA expression data from GEO, gEAR, and SHIELD databases. *Adamts1, Mpdz, Mvd*, and *Sez6* were all expressed in the inner ear during the embryonic, postnatal, and adult stages, suggesting an involvement of the encoded proteins in the development and maintenance of the cochlear sensory epithelium. Our analysis showed widespread expression of *Adamts1*, *Mpdz*, and *Mvd* in the cochlear epithelium while *Sez6* expression is localized to the hair cells. Immunohistochemistry results confirmed the expression of ADAMTS1, MPDZ, and SEZ6 in the hair cells. Additionally, it also demonstrated that MVD is expressed in the spiral ganglion cells, which play an integral part in the perception of sound.

ADAMTS1, is a member of the matrix metalloproteinases (MMPs) family. ADAMTS1 and members of this family of metalloproteinases are secreted and either bind extracellular matrix components or attach to the cell surface through particular regulatory mechanisms. *ADAMTS1* is responsible for mediating acute regulated tissue remodeling processes that occur in development [22]. We identified a missense variant [c.403T>G:p.(Ser135Ala)] in *ADAMTS1* which segregated with HI, is predicted to be deleterious and may affect the solvent accessible surface area of the mutant protein. Our analysis shows the presence of *Adamts1* in the inner ear from as early as embryonic stage E12 to the adult stages indicating a role in both development and functioning of the inner ear. A study of mice MMPs has revealed that these proteins are involved in the modulation of cochlear responses to acoustic trauma in rats. *Adamts1* was found to be upregulated after exposure to noise in the mouse sensory epithelium [23], suggesting a role for *Adamts1* in the response to acoustic trauma.

MPDZ, the multidomain PDZ domain protein (MPDZ/MUPP1) contains 13 PDZ domains, and mainly serves as a mediator of multiple protein–protein interactions as a scaffold protein [24]. The variant we identified, c.2324C>T:p.(Pro775Leu), is predicted to result in considerably destabilizing conformational changes to the protein. *MPDZ* has previously been associated with congenital hydrocephalus with and without brain or eye anomalies, posteriorly rotated ears, and sensorineural HI [25], however it has not been implicated in NSHI. *Mpdz*^*em1(IMPC)J*^ homozygous mice, generated and phenotyped by the International Mouse Phenotyping Consortium have mild hearing loss that affects all frequencies [reduced auditory brainstem response (*p* = 4.93 × 10^–12^)][26]. MUPP-1/MPDZ bind integral membrane proteins like claudins and link the tight junction (TJ) to the actin cytoskeleton [27, 28]. In the organ of Corti, TJs of reticular lamina (which consists of a mixture of sensory hair and supporting cells) separate K^+^-rich endolymph and Na^+^-rich perilymph [27], and also gives structural support to auditory neuroepithelium. Our analyses showed that Mpdz was highly expressed in the cochlear epithelium including cochlear hair cells, utricle hair cells, OHCs, IHCs, surrounding hair cells to the cochlea and utricle and cytoplasmic region of the hair cells. Various studies have identified high concentration of MPDZ in TJs due to possible interactions with other membrane associated proteins [29]. Some of these partner molecules are those that support the TJs in the inner ear but whether these functional associations with Mpdz are also maintained in the inner ear has not been studied.

The mevalonate pyrophosphate decarboxylase enzyme encoded by *MVD* plays a pivotal role in the pre-squalene stage of the mevalonate pathway that performs the first step in the biosynthesis of isoprenes/cholesterol [30]. Cholesterol and isoprenoid compounds are essential for the development and function of the neurons in the central nervous system [31]. The identified *MVD* variant, c.1136C>A:p.(Pro379His) may affect the interactions of the mutant protein. Defects in both pre and post-squalene pathways of cholesterol synthesis are associated with neurodegenerative and dermatological conditions [32]. It has also been suggested that hypercholesterolemia may be linked to HI in both animal models and humans [33]. Cholesterol plays a role in the regulation of auditory calcium and calcium-activated potassium channels augmenting the growing evidence that it is a strategic factor in auditory physiology [34]. Ion channels are critical for the spiral ganglion neurons in establishing the differences in response properties that are crucial for normal hearing [35], and our immunohistochemistry results show, the Mvd is preferentially expressed in the spiral ganglion cells. In addition, variations in cochlear cholesterol levels modulate the amplitude of distortion product otoacoustic emissions, consistent with changes in electromotility [36]. Further, the cholesterol levels in OHCs are also linked to the capacitance, electromotility and electrical signature associated with the function of prestin, a transmembrane protein critical to sensitive hearing and OHC electromotility in mammals [37]. RNA expression analysis indicated that *Mvd* exhibits a widespread expression in the cochlear epithelium, IHCs, OHCs, and surrounding hair cells to the utricle and cochlea.

The *SEZ6* gene encodes the seizure-related 6 protein and has a restricted expression profile. It is primarily concentrated in the brain (in the somatodendritic surface of neurons) [38], and is considered to be involved in synaptic development and function [39]. In our analysis we found that in the inner ear cells SEZ6 has a highly localized expression in the hair, deiter’s, pillar, and outer sulcus cells, stereocilia and cytoplasm of OHCs. The expression was highly varied across developmental stages with a higher expression levels during the postnatal than the embryonic stage. In mice, the *Sez6* family has been shown to be involved in modulation of synapse numbers, synaptic plasticity, and dendrite morphology in the cortex and hippocampus and neuronal connectivity in the cerebellum. It was also observed that the loss of the gene results in impaired cognition, motor learning, and motor functions. To date, no studies have reported *SEZ6* variants to be involved in HI although genetic variants of SEZ6 family proteins have been associated or are potential candidates for several neurodevelopmental disorders [40]. The c.2092G>A:p.(Val698Ile) is predicted to result in destabilizing changes without totally damaging the structure of the protein. It has been observed that SEZ6 acts as a trafficking factor for GluK2/K3 [41], which are part of the KAR family (GluK1, GluK2, GluK3, and GluK5). The KARs are a group of ionotropic glutamate receptors found to be localized in adult OHC and IHC afferent synapses and terminals [42], which function coordinately in transferring acoustic signals across the synapses. In our analyses, we have observed that *Sez6* has higher expression levels in the postnatal stages and is expressed in both the cytoplasm and stereocilia of hair cells, supporting the role of *Sez6* in mediating acoustic signals. Taken together, this evidence implies a role of SEZ6 in neurotransmission in the hair cells, which is supported by its localized expression pattern in the inner ear hair cells.

In conclusion, we identified four candidate genes for ARNSHI, that all show expression in the developing and/or adult inner ear. The identified variants are predicted to lead to structural, conformational, and interaction changes to the protein. The variants segregate with profound prelingual bilateral sensorineural HI and for three of the variants there is significant evidence of linkage providing additional support to their role in ARNSHI. Although for the *MPDZ* variant only suggestive linkage was observed, a mouse model with HI further supports its involvement in NSHI etiology. The identification of ARNSHI candidates, *ADAMTS1, MPDZ, MVD*, and *SEZ6* aids in further expanding our knowledge of HI etiology.

## Supplementary information

Supplementary Material

Supplementary Table 1

Supplementary Table 2

## Data Availability

The variants have been submitted to ClinVar database [Accession numbers: SCV001478241 (*ADAMTS1* [c.403T>G:p.(Ser135Ala)]), SCV001478242 (*MPDZ* [c.2324C>T:p.(Pro775Leu)]), SCV001478243 (*MVD* [c.1136C>A:p.(Pro379His)]), and SCV001478244 (*SEZ6* [c.2092G>A:p.(Val698Ile)])].
