# Supplementary material for: ADAMTS1, MPDZ, MVD, and SEZ6: candidate genes for autosomal recessive nonsyndromic hearing impairment
Source: Eur J Hum Genet. 2021 Jun 16;30(1):22–33. doi: 10.1038/s41431-021-00913-x (PMC8738740; doi:10.1038/s41431-021-00913-x)
Supplement: Supplementary file 1 — Supplementary Material [file 41431_2021_913_MOESM1_ESM.pdf]

**Description of Supplementary Information**

Supplementary information contains Acknowledgements, 11 Figures, Web resources, and Tables in separate excel files.

**Acknowledgements**

**Supplementary Figures 1-11:** Expression of candidate genes in the mouse inner ear

**Web resources**

**Supplementary Tables 1-2:** Details on segregating and non-segregating variants identified in the families under study

## Supplemental Acknowledgements

We also thank the following who provided sequencing at the University of Washington Center for Mendelian Genomics (UW-CMG): Michael J. Bamshad[1,2], Suzanne M. Leal[3], Deborah A. Nickerson[1], Peter Anderson[1], Tamara J. Bacus[1], Elizabeth E. Blue[1], Kati J. Buckingham[1], Jessica X. Chong[1], Diana Cornejo Sánchez[3], Colleen P. Davis[1], Christian D. Frazar[1], Danielle Giroux[1], William W. Gordon[1], Martha Horike-Pyne[1], Jameson R. Hurless[1], Gail P. Jarvik[1], Eric Johanson[1], J. Thomas Kolar[1], Melissa P. MacMillan[1], Colby T. Marvin[1], Sean McGee[1], Daniel J. McGoldrick[1], Betselote Mekonnen[1], Patrick M. Nielsen[1], Karynne Patterson[1], Benjamin Pelle[1], Aparna Radhakrishnan[1], Matthew A. Richardson[1], Gwendolin T. Roote[1], Erica L. Ryke[1], Isabelle Schrauwen[3], Kathryn M. Shively[1], Joshua D. Smith[1], Monica Tackett[1], Machiko S. Threlkeld[1], Gao Wang[3], Jeffrey M. Weiss[1], Marsha M. Wheeler[1], Qian Yi[1], Jordan E. Zeiger[1], and Xiaohong Zhang[1] Affiliations: [1]University of Washington; [2]Seattle Children's Hospital; and [3]Columbia University. UW-CMG was funded by the National Human Genome Research Institute and the National Heart, Lung and Blood Institute grant HG006493 to Michael J. Bamshad[1,2], Suzanne M. Leal[3], Deborah A. Nickerson[1].

## Supplementary Figures

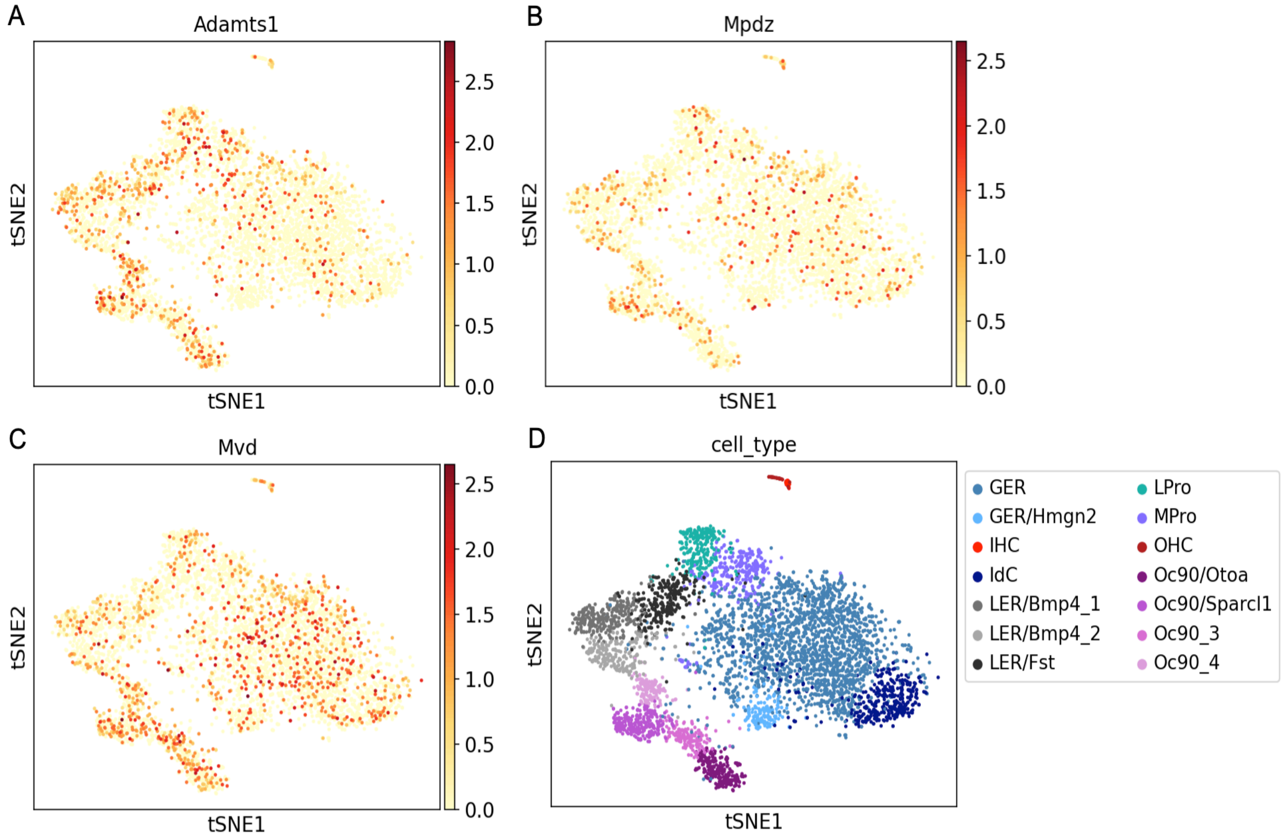


**Supplementary Figure 1: tSNE plots of the cochlear floor cells at E14 stage illustrating the expression of *Adamts1, Mpdz,* and *Mvd* genes.**

Identified genes are expressed in the cochlear floor cells of the mouse inner ear at the E14 stage. The dataset consists of cells from the cochlear floor epithelia duct of E14 litters of two wild type timed-pregnant CD-1 females. Each litter had 10-12 pups of both sexes. Panel A. *Adamts1*, Panel B. *Mpdz*, Panel C. *Mvd*, and Panel D. Reference panel for panels A-C illustrating the location of each specific cell type. Tissue codes: GER (Greater Epithelial Ridge), GER/Hmgn2 (Greater Epithelial Ridge expressing Hmgn2), IHC (Inner Hair Cells), IdC (Interdental Cells), LER/Bmp4_1*, LER/Bmp4_2* (Lesser Epithelial Ridge cells expressing Bmp4), LER/Fst (Lesser Epithelial Ridge cells expression Fst), LPro (Lateral Prosensory Cells), MPro (Medial

Prosensory Cells), OHC (Outer Hair Cells), Oc90/Otoa # (Cells expressing Oc90 and Otoa), Oc90/Sparcl1 # (Cells expressing Oc90 and Sparcl1), Oc90_3 #* (Cells expressing Oc90), Oc90_4 #* (Cells expressing Oc90) (* Distinct clusters identified by Seurat v2.0, some expressing similar markers like Bmp4 or Oc90, # Oc90+ cells may be early Reissner's membrane). The scale bar in panel A-C represents gene expression ranging from low-yellow to high-red based on log transformed, normalized, and scaled for sequencing depth expression data.

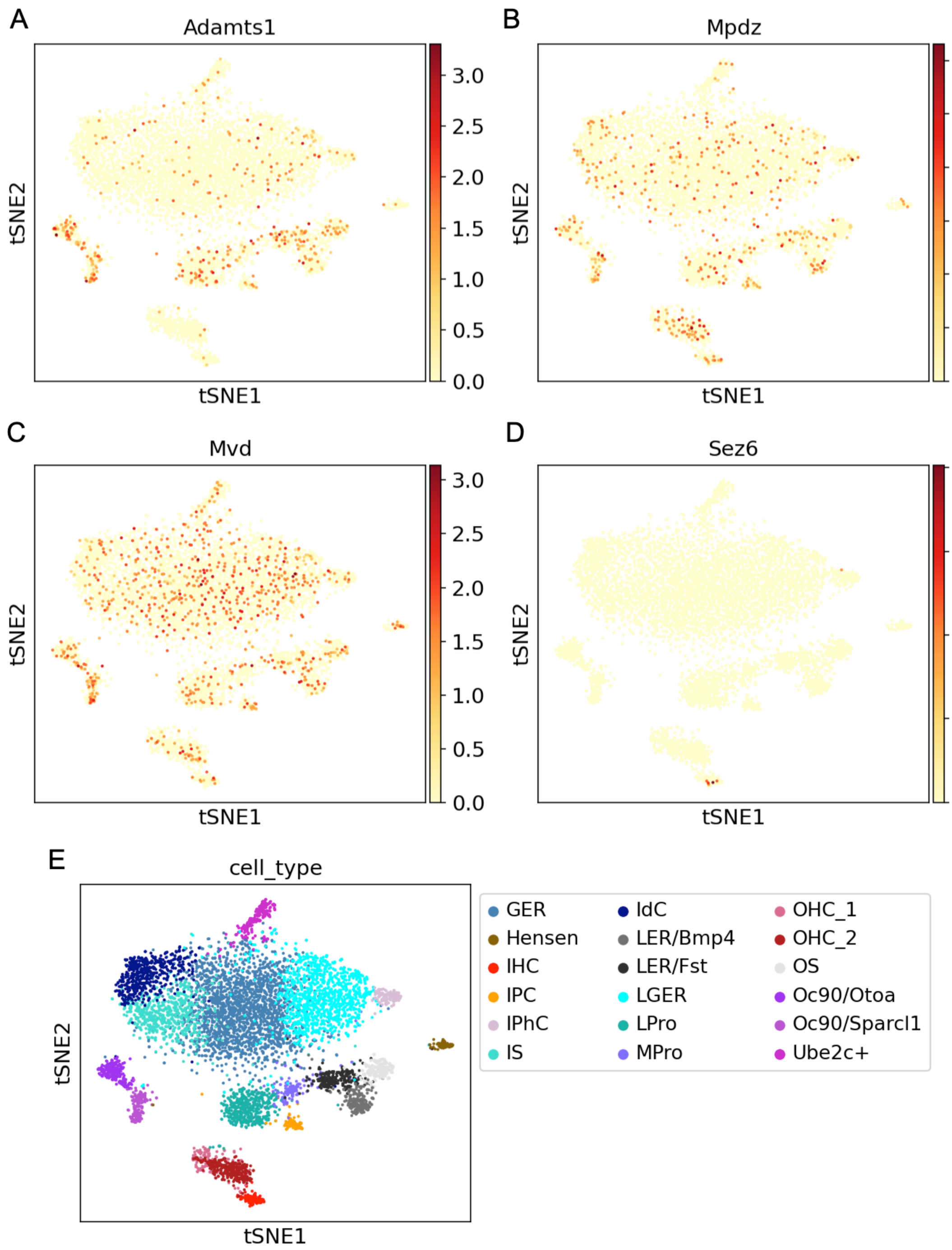


**Supplementary Figure 2: tSNE plots of the cochlear floor cells at E16 stage illustrating the expression of *Adamts1, Mpdz, Mvd,* and *Sez6* genes.**

Identified genes are expressed in the cochlear floor cells of the mouse inner ear at the E16 stage. The dataset comprises cells from the cochlear floor epithelia duct of E16

stage litters of three wild type timed-pregnant CD-1 females. Each litter had 10-12 pups of both sexes. Panel A. *Adamts1*, Panel B. *Mpdz*, Panel C. *Mvd*, Panel D. *Sez6*, and Panel E. Reference panel for panels A-D illustrating the location and cell type. Tissue codes: GER (Greater Epithelial Ridge), Hensen (Hensen Cells), IHC (Inner Hair Cells), IPC (Inner Pillar Cells), IPhC (Inner Phalangeal Cells), IS (Inner Sulcus Cells), IdC (Interdental Cells), LER/Bmp4 (Lesser Epithelial Ridge cells expressing Bmp4), LER/Fst (Lesser Epithelial Ridge cells expression Fst), LGER (Lateral Greater Epithelial Ridge Cells), LPro (Lateral Prosensory Cells), MPro (Medial Prosensory Cells), OHC_1 (More mature developing outer hair cells), OHC_2 (Less mature developing outer hair cells), OS (Outer Sulcus Cells), Oc90/Otoa (Cells expressing Oc90 and Otoa), (Oc90/Sparcl1) Cells expressing Oc90 and Sparcl1, Ube2c+ (Unannotated cells expressing Ube2c+). The scale bar in panel A-D represents gene expression ranging from low-yellow to high-red based on log transformed, normalized, and scaled for sequencing depth expression data.

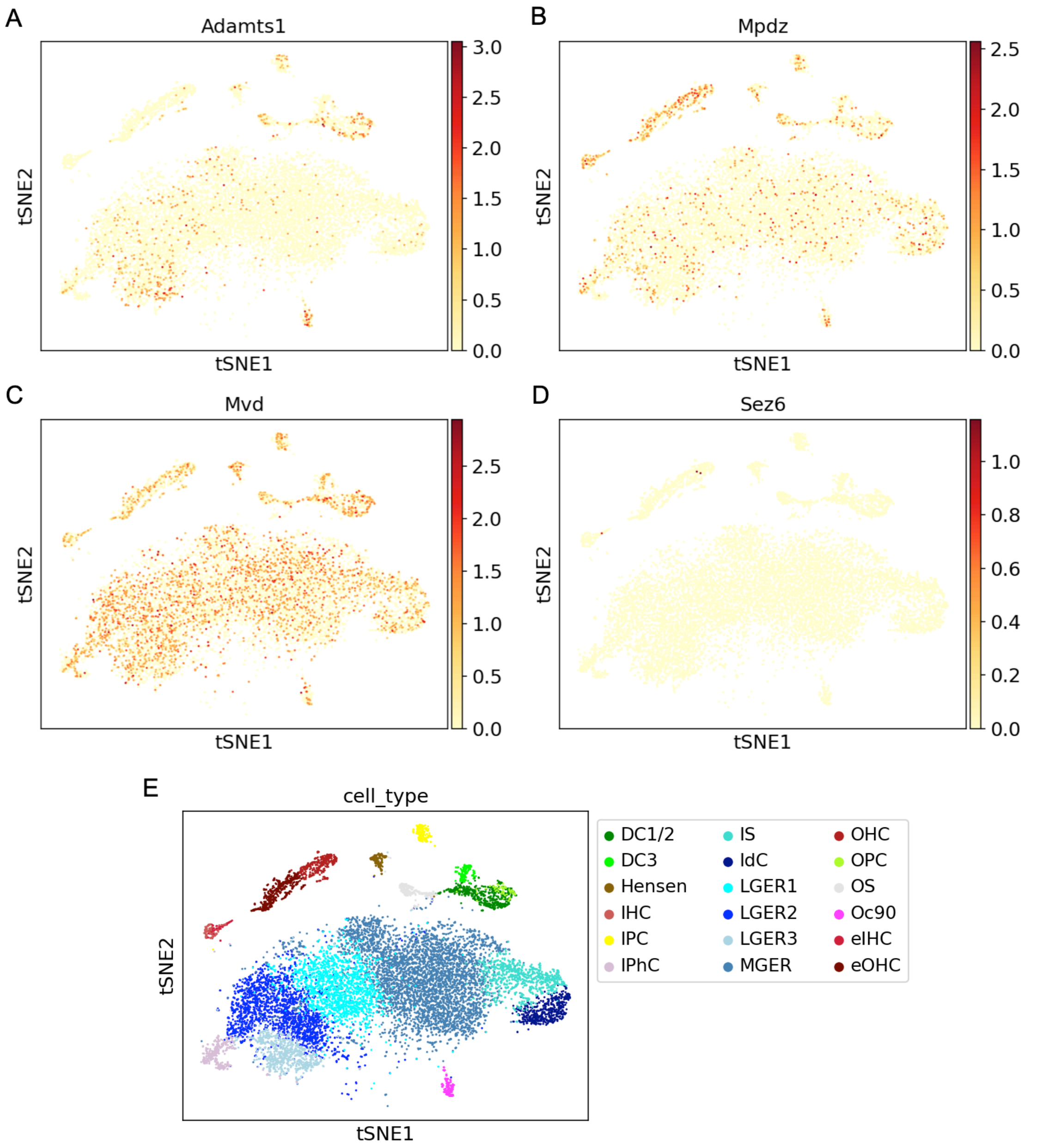


**Supplementary Figure 3: tSNE plots of the cochlear floor cells at P1 stage illustrating the expression of *Adamts1, Mpdz, Mvd,* and *Sez6* genes.**

Identified genes are expressed in the cochlear floor cells of the inner ear of the mouse at P1 stage. This dataset consists of cells obtained from the cochlear floor epithelia duct of ~20-32 P1 CD-1 pups of both sexes. Panel A. *Adamts1*, Panel B. *Mpdz*, Panel C. *Mvd*, Panel D. *Sez6*, and Panel E. Reference panel for panels A-D illustrating the location and

cell type. Tissue codes: Developing supporting cells- DC1/2 (Deiters' cells rows 1 and 2), DC3 (Deiters' cells row 3), Hensen (Hensen's cells), IPC (inner pillar cells), IPhC (inner phalangeal cells/border cells), IS (inner sulcus cells), IdC (interdental cells), OPC (outer pillar cells), OS (Outer sulcus cells), Oc90 ($OC90^+$ cells). Developing greater epithelial ridge cells- LGER1, LGER2, LGER3, MGER. Developing sensory cells- IHC (Inner hair cells), OHC (Outer hair cells), eIHC, eOHC. The scale bar in panel A-D represents gene expression ranging from low-yellow to high-red based on log transformed, normalized, and scaled for sequencing depth expression data.

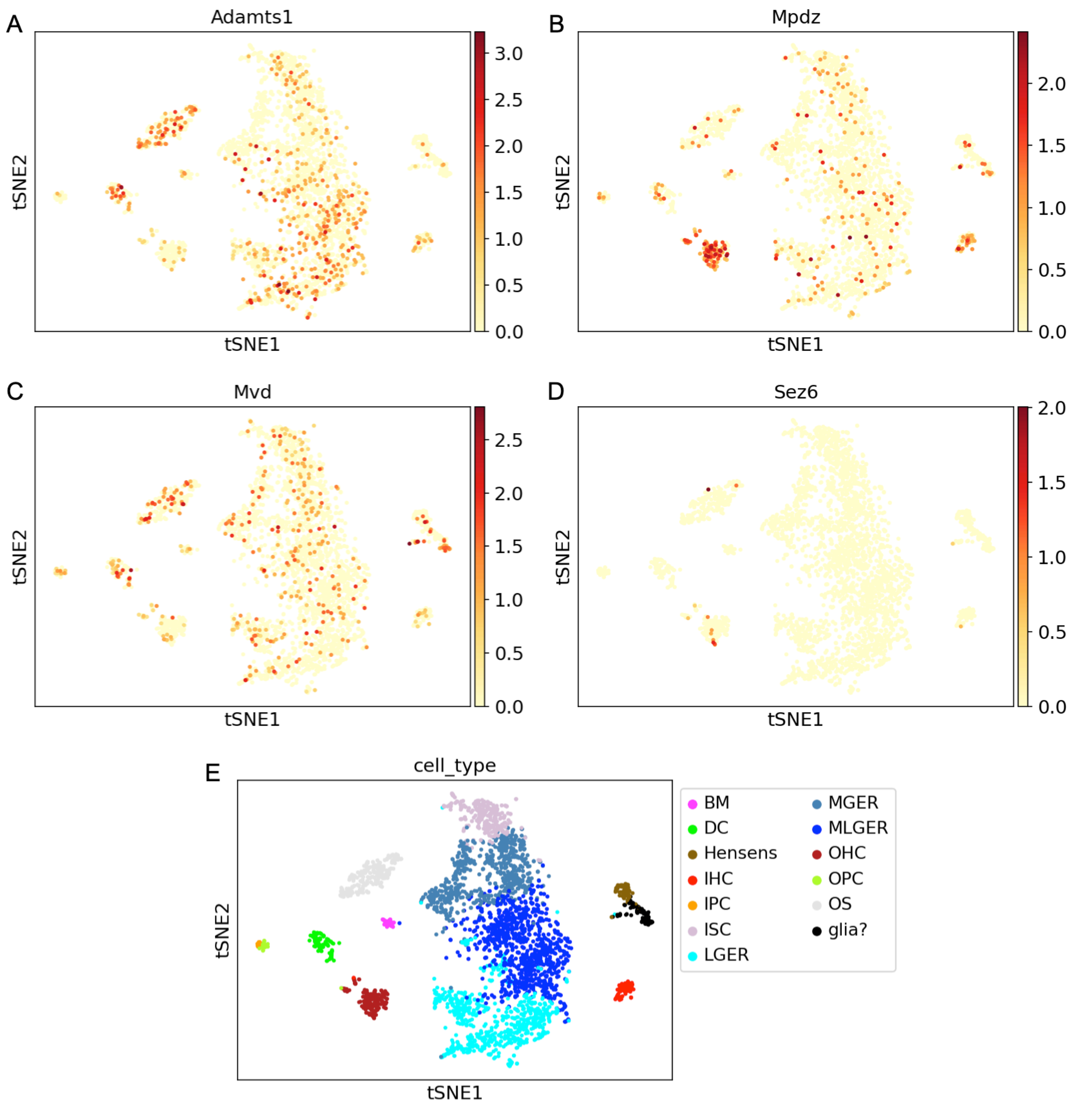


**Supplementary Figure 4: tSNE plots of the cochlear floor cells at P7 stage illustrating the expression of *Adamts1, Mpdz*, *Mvd*, and *Sez6* genes.**

Identified genes are expressed in the cochlear cells of the inner ear of the mouse at the P7 stage. The dataset contains cells obtained from the cochlear floor epithelia duct of ~15-24 P7 CD-1 pups of both sexes. Panel A. *Adamts1,* Panel B. *Mpdz*, Panel C. *Mvd*, Panel D. *Sez6*, and Panel E. Reference panel for panels A-D illustrating the location and cell. Tissue codes: BM (Basilar Membrane cells), DC (Deiters' cells), Hensen (Hensen's cells), IHC (Inner Hair Cells), IPC (Inner Pillar Cells), ISC (Inner Sulcus

Cells), LGER (Lateral Greater Epithelial Ridge Cells), MGER (Medial Greater Epithelial Ridge Cells), MLGER (Medial Lateral Greater Epithelial Ridge Cells), OHC (Outer hair cells), OPC (outer pillar cells), OS (Outer sulcus cells), Glia (Glial cells). The scale bar in panel A-D represents gene expression ranging from low-yellow to high-red based on log transformed, normalized, and scaled for sequencing depth expression data.

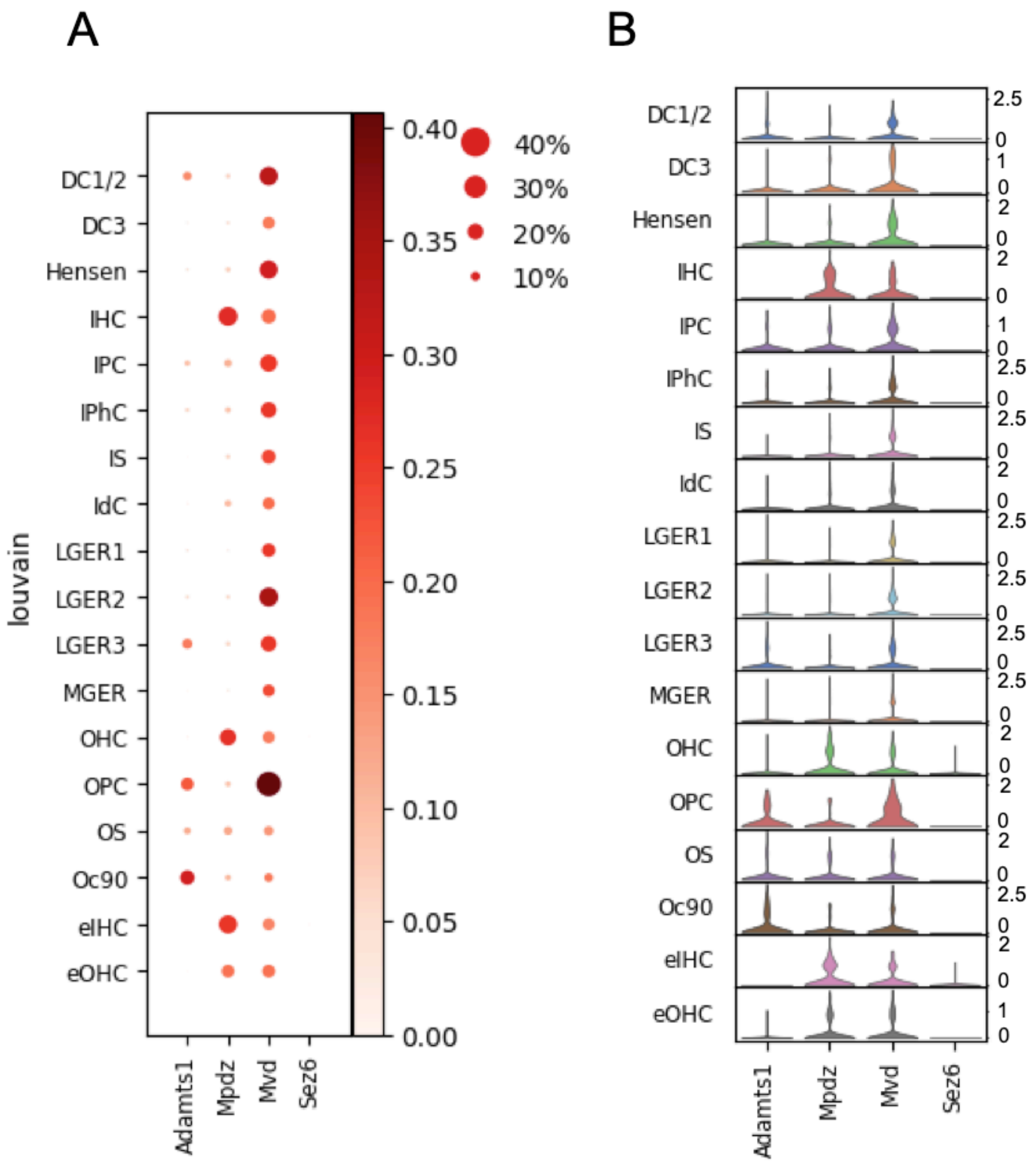


**Supplementary Figure 5: Comparative gene expression of the four candidate genes in different cell clusters in cochlear epithelia at the P1 stage.**

The dataset consists of cells obtained from the cochlear floor epithelia duct of P1 stage CD-1 pups of both sexes.

Tissue codes: Developing supporting cells- DC1/2 (Deiters' cells rows 1 and 2), DC3 (Deiters' cells row 3), Hensen (Hensen's cells), IPC (inner pillar cells), IPhC (inner phalangeal cells/border cells), IS (inner sulcus cells), IdC (interdental cells), OPC (outer

pillar cells), OS (Outer sulcus cells), Oc90 ($OC90^+$ cells). Developing greater epithelial ridge cells- LGER1, LGER2, LGER3, and MGER. Developing sensory cells- IHC (Inner hair cells), OHC (Outer hair cells), eIHC (Less mature developing inner hair cells, eOHC (Less mature developing outer hair cells). Scale represents log transformed, normalized, and scaled for sequencing depth expression data.

**Panel A.** Dot plot displays expression levels of *Adamts1*, *Mpdz*, *Mvd*, and *Sez6*. Expression levels of the genes are represented by color intensity with the highest level of expression displayed in dark red (as indicated in the scale bar). Dot size represents the proportion of cells expressing the particular gene.

**Panel B.** Violin plots display the relative expression levels of *Adamts1*, *Mpdz*, *Mvd*, and *Sez6* in each cluster of cells. *Sez6* expression is observed mainly in IHCs and OHCs.

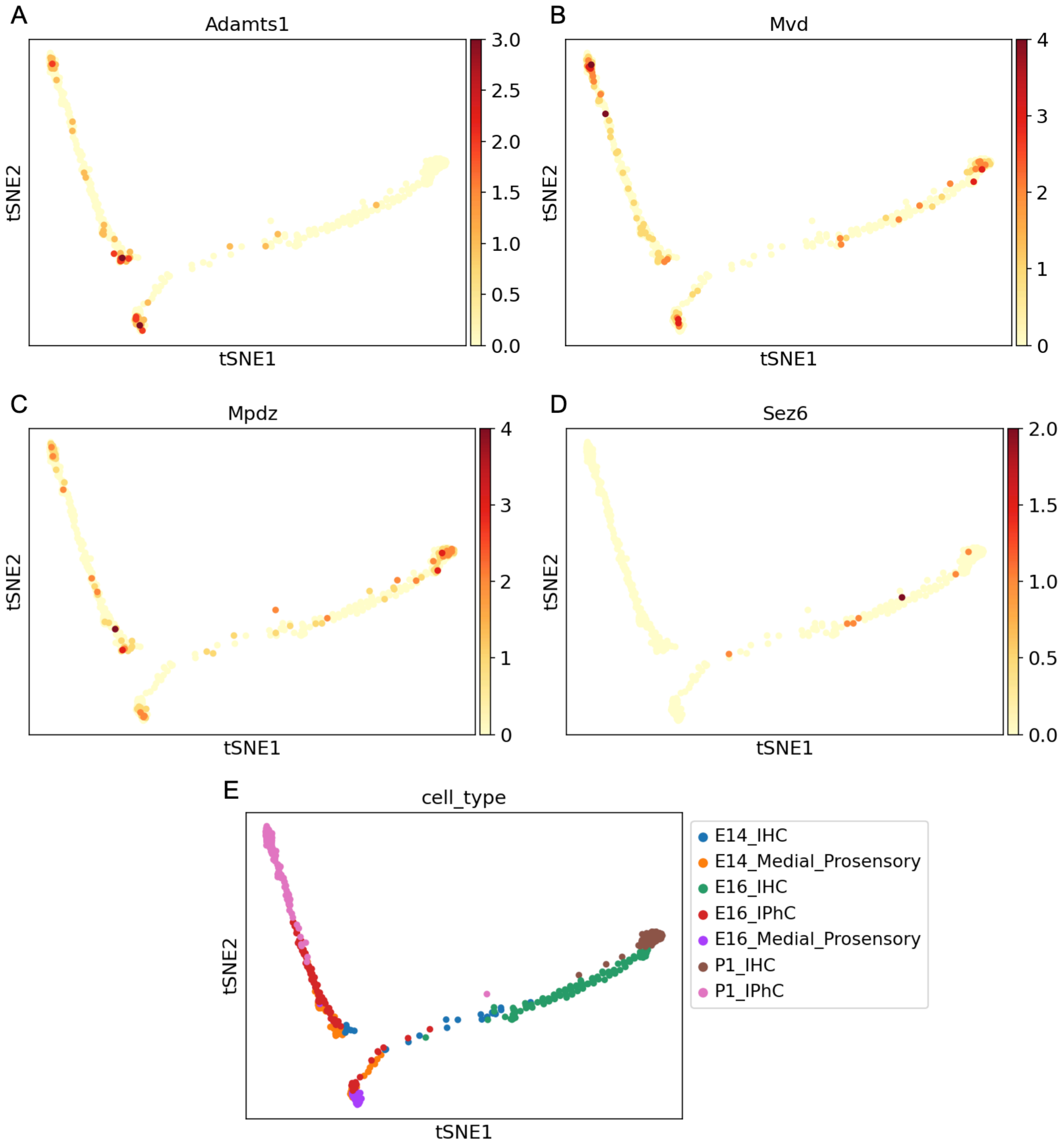


**Supplementary Figure 6: tSNE plots of inner hair cells at E14, E16, and P1 stages illustrating the expression of *Adamts1, Mpdz*, *Mvd*, and *Sez6* genes.**

Expression of the four candidate genes in the mouse inner hair cells at the E14, E16, and P1 stages. This dataset comprises hair cells and supporting cells obtained from the cochlear floor epithelia duct. mRNA was collected from E14 litters from two female pregnant CD-1 mice, E16 litters from three female pregnant CD-1 mice, and ~20-32 P1 CD-1 pups.

Panel A. *Adamts1,* Panel B. *Mpdz*, Panel C. *Mvd*, Panel D. *Sez6*, and Panel E. Reference panel for panels A-D illustrating the location and cell type. Tissue codes: E14_IHC (Early developing inner hair cells, E14), E14_Medial_Prosensory (Developing medial prosensory cells, E14), E16_IHC (Developing inner hair cells, E16), E16_IPhC (Developing inner phalangeal cells, E16), E16_Medial_Prosensory (Developing medial prosensory cells, E16), P1_IHC (Developing inner hair cells, P1), P1_IPhC (Developing inner phalangeal cells, P1). The scale bar in panel A-D represents gene expression ranging from low-yellow to high-red based on log transformed, normalized, and scaled for sequencing depth expression data.

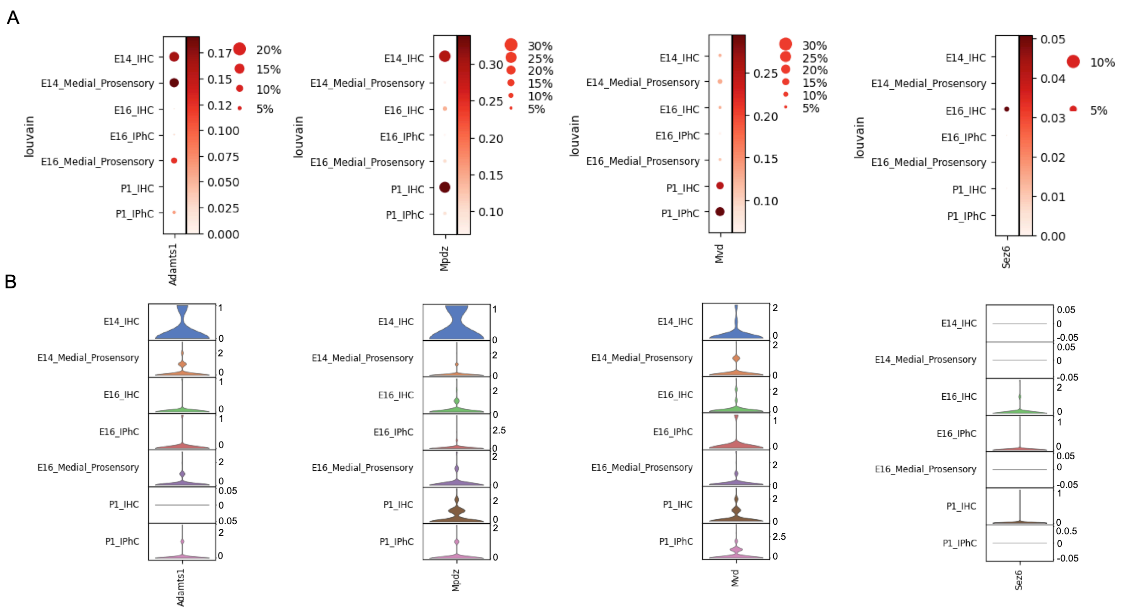


**Supplementary Figure 7: Comparative gene expression of candidate genes in the inner hair cells at stages E14, E16, and P1.**

The dataset contains hair cells and supporting cells from the cochlear floor epithelia duct. mRNA was collected at the E14 litter from two female pregnant CD-1 mice and E16 stage litter from three female pregnant CD-1 mice and ~20-32 P1 CD-1 pups.

Tissue codes: E14_IHC (Early developing outer hair cells, E14), E14_Medial_Prosensory (Developing medial prosensory cells, E14), E16_IHC (Developing inner hair cells, E16), E16_Medial_Prosensory (Developing medial prosensory cells, E16), P1_IHC (Developing inner hair cells, P1), P1_IPhC (Developing inner phalangeal cells, P1). The scale represents log transformed, normalized, and scaled for sequencing depth expression data.

**Panel A.** The dot plots display expression levels for *Adamts1, Mpdz*, *Mvd*, and *Sez6*. Low to high expression of the genes are represented by color intensity which increases towards dark red (as indicated in the scale bar) for the highest expression level. The dot size represents the proportion of cells expressing the gene.

**Panel B.** Violin plots displaying relative expression levels of *Adamts1*, *Mpdz*, *Mvd*, and *Sez6* in each cluster of cells.

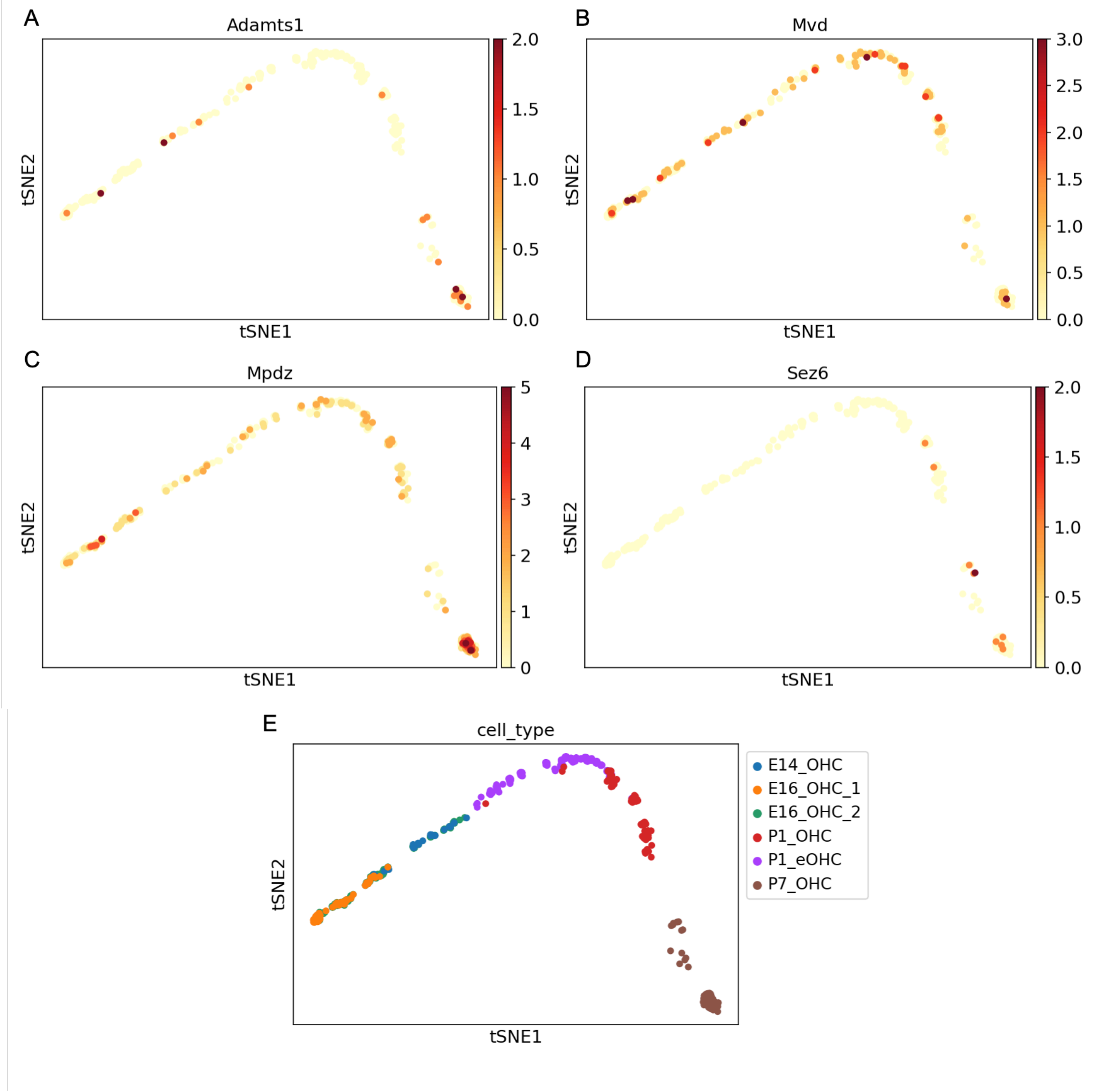


**Supplementary Figure 8: tSNE plots of the outer hair cells at E14, E16, P1 and P7 stages illustrating the expression of *Adamts1, Mpdz*, *Mvd*, and *Sez6* genes.**

Expression of the four candidate genes in the mouse outer hair cells at the E14, E16, P1, and P7 stages. This dataset contains outer hair cells from the cochlear floor epithelia duct. mRNA was collected from E14 litter from two female pregnant CD-1 mice, E16 litter from three female pregnant CD-1 mice, ~20-32 P1 CD1 pups, and ~15-24 P7 CD-1 pups of both sexes. Panel A. *Adamts1*, Panel B. *Mpdz*, Panel C. *Mvd*, Panel D. *Sez6*, and Panel E. Reference panel for panels A-D illustrating the location and cell type.

Tissue codes: E14_OHC (Early developing outer hair cells, E14), E16_OHC_1 (More mature developing outer hair cells, E16), E16_OHC_2 (Less mature developing outer hair cells, E16), P1_OHC (More mature developing outer hair cells, P1), P1_eOHC (Less mature developing outer hair cells, P1), P7_OHC (Developing outer hair cells, P7). The scale bar in panel A-D represents gene expression ranging from low-yellow to high-red based on log transformed, normalized, and scaled for sequencing depth expression data.

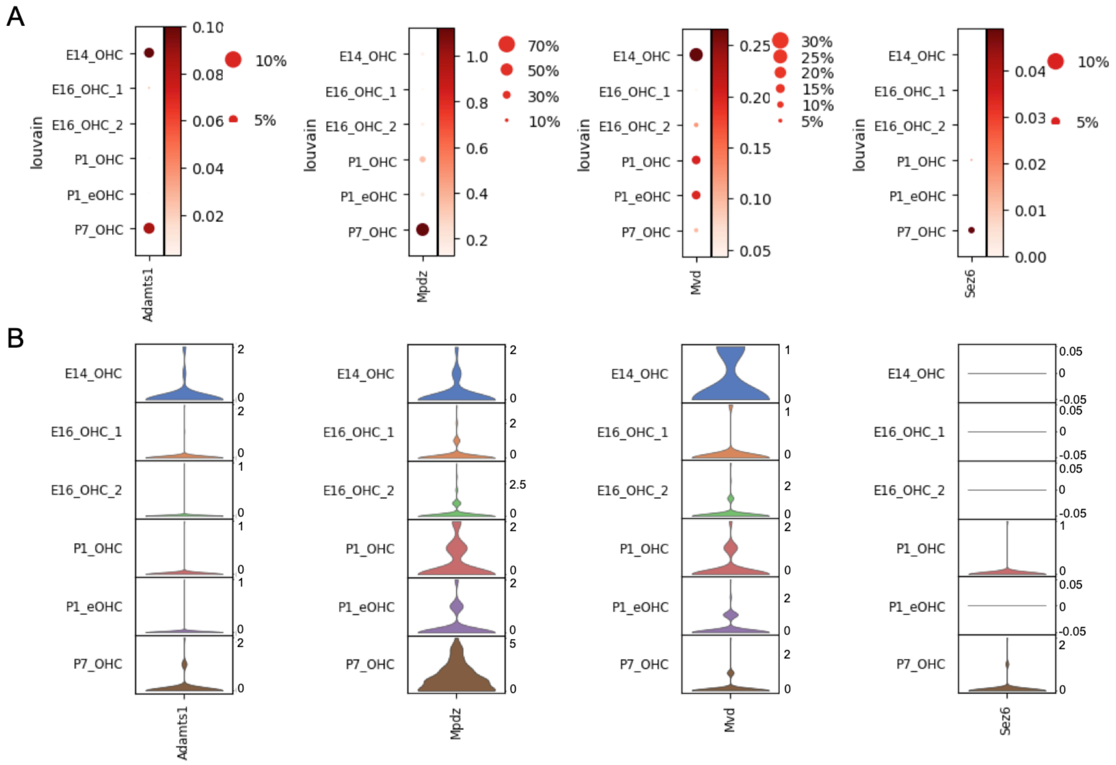


**Supplementary Figure 9: Comparative gene expression of candidate genes in outer hair cells at stages E14, E16, P1 and P7.**

The dataset contains outer hair cells from cochlear floor epithelia duct. mRNA was collected from E14 litter from two female pregnant CD-1 mice, E16 litter from three

female pregnant CD-1 mice, ~20-32 P1 CD-1 pups, and ~15-24 P7 CD-1 pups of both sexes. Tissue code: E14_OHC (Early developing outer hair cells, E14), E16_OHC_1 (More mature developing outer hair cells, E16), E16_OHC_2 (Less mature developing outer hair cells, E16), P1_OHC (More mature developing outer hair cells, P1), P1_eOHC (Less mature developing outer hair cells, P1), P7_OHC (Developing outer hair cells, P7). The scale represents log transformed, normalized, and scaled for sequencing depth expression data.

**Panel A.** The dot plots display expression levels of *Adamts1, Mpdz*, *Mvd*, and *Sez6*. Expression of the genes is represented by color intensity with dark red (as indicated in the scale bar) having the highest expression levels. Dot size represents the proportion of cells expressing the particular gene.

**Panel B.** Violin plot displaying the relative expression of *Adamts1, Mpdz, Mvd* and *Sez6* in each cluster of cells

*The figures 1 to 9 are based on the single cell expression data obtained from and created through the gEAR analysis suite. The scale represents log transformed, normalized, and scaled for sequencing depth expression data.*

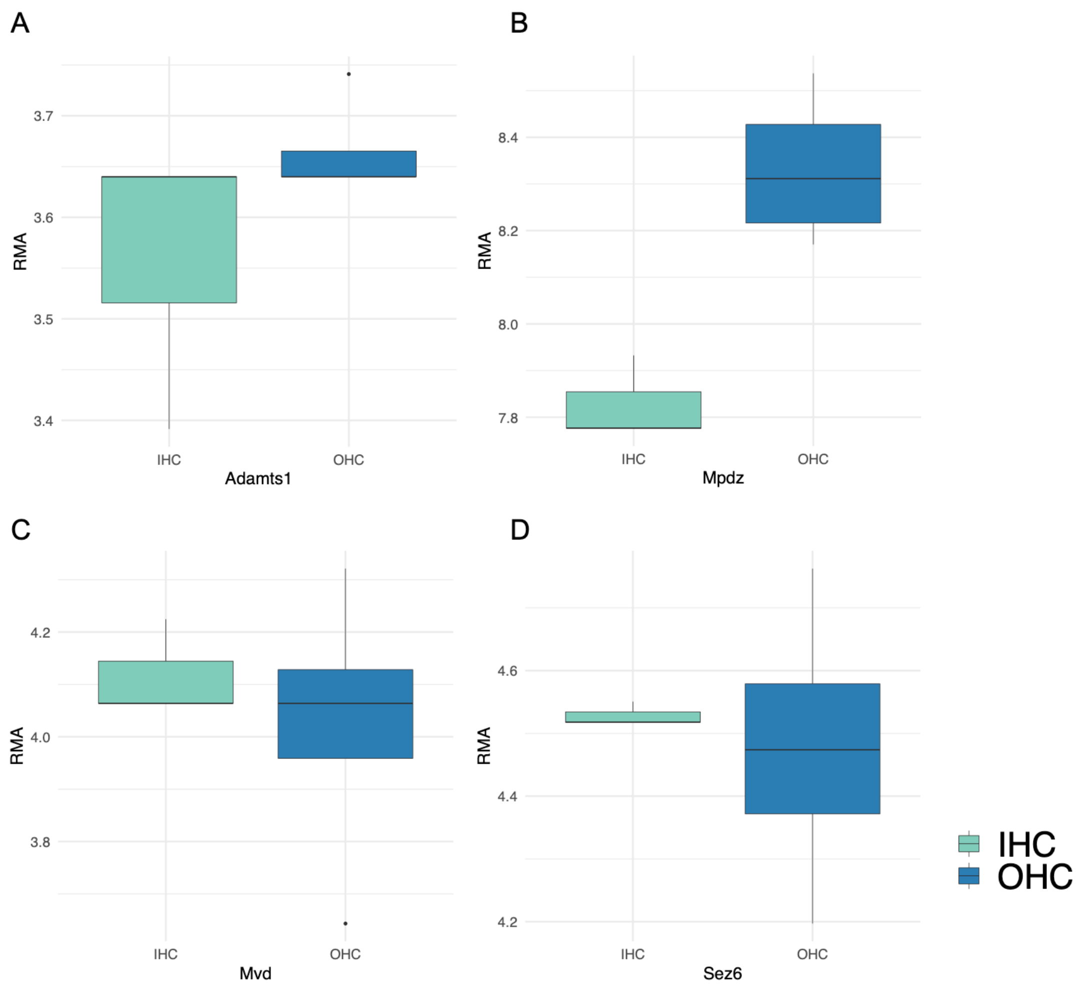


**Supplementary Figure 10: Comparison of RNA expression of the candidate genes between cochlear IHCs and OHCs in adult mice.**

The expression of candidate genes in IHCs and OHCs of adult CBA/J mice (25-35 days old) using processed expression data from the study GSE56866 in the GEO database. Panel A. *Adamts1*, Panel B. *Mpdz*, Panel C. *Mvd*, and Panel D. *Sez6.* The genes were expressed in adult mouse cochlear hair cells. The y-axis scale bar represents the normalized RMA (Robust multiarray average) values.

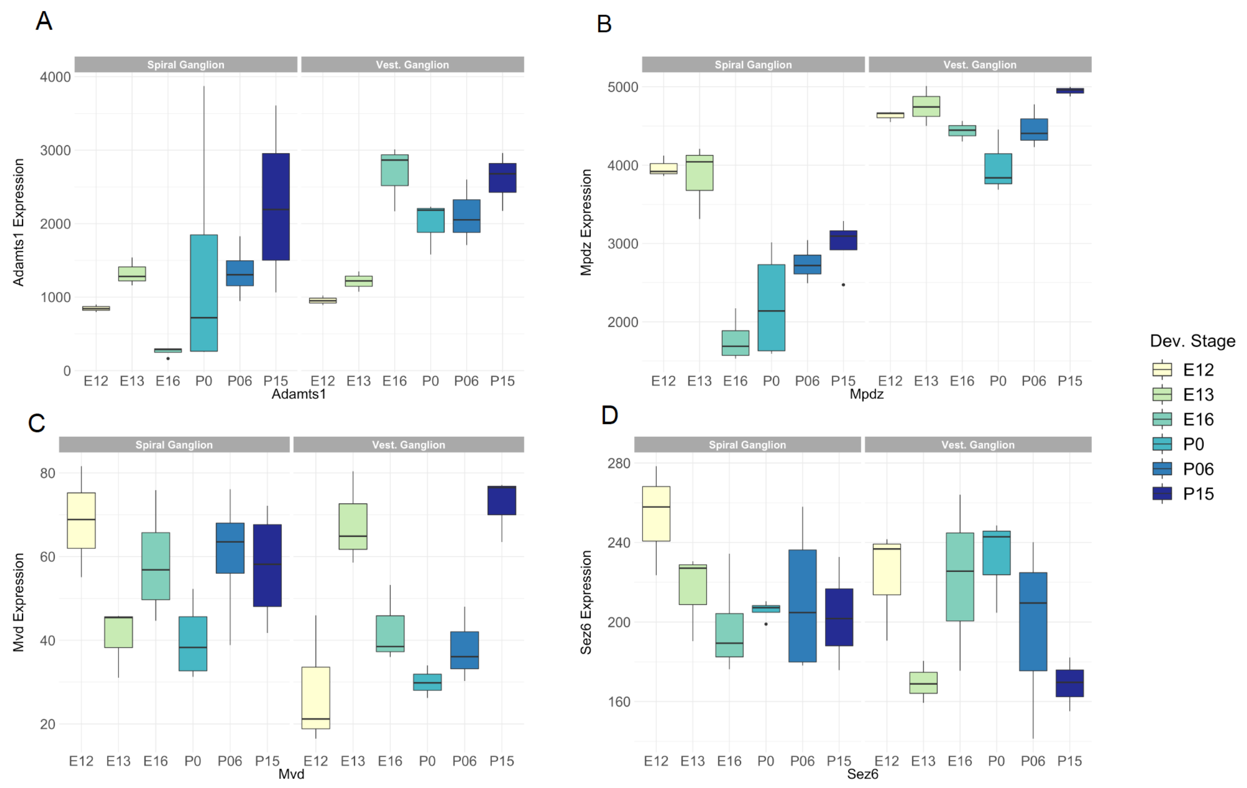


**Supplementary Figure 11: Expression of the four candidate genes in spiral and vestibular ganglion neurons during mouse development.**

Data from RNA micro-arrays of gene expression based on perfect match and mismatch probe differences (PM/MM) in spiral ganglion neurons and vestibular ganglion neurons from mice. Data collection occurred at six developmental stages: E12, E13, E16, P0, P06, and P15.

**Panel A.** In the spiral ganglion Adamts1 expression is low during E13-E16, it increases in the vestibular ganglion to its highest expression level at E16. Adamts1 expression remains high during P0-P15 in both spiral ganglion and vestibular ganglion.

**Panel B.** Data shows that *Mpdz* expression increases during E12-E13 after which it decreases slightly. But this is followed by an increase at the P15 stage in both the spiral and vestibular ganglion neurons.

**Panel C.** *Mvd* is expressed in all stages from E12-P15 in both spiral ganglion and vestibular ganglion.

**Panel D.** *Sez6* expression is higher at E13 than at E16-P0 stages in spiral ganglion. In the vestibular ganglion the highest expression is seen in E12, E16 and P0 stages.

*The data was obtained from SHIELD (Shared Harvard Inner-Ear Laboratory Database).*

**Supplementary Web resources**

ANNOVAR, https://doc-openbio.readthedocs.io/projects/annovar/en/latest/

Bio Mart, http://www.biomart.org

Burrows-Wheeler Aligner, http://bio-bwa.sourceforge.net/

ClinVar, https://www.ncbi.nlm.nih.gov/clinvar/

Centers for Disease Control and Prevention,

https://www.cdc.gov/ncbddd/hearingloss/genetics.html

Combined Annotation Dependent Depletion (CADD), http://cadd.gs.washington.edu/

dbSNFP, https://sites.google.com/site/jpopgen/dbNSFP

dbSNP, https://www.ncbi.nlm.nih.gov/projects/SNP/

gEAR, https://umgear.org/

Genome Aggregation Database (gnomAD), http://gnomad.broadinstitute.org/

Genome Analysis Toolkit (GATK), https://software.broadinstitute.org/gatk/

Genomic Evolutionary Rate Profiling (GERP),

http://mendel.stanford.edu/SidowLab/downloads/gerp/

Greater Middle East (GME) Variome Project, http://igm.ucsd.edu/gme

Hereditary Hearing Loss Homepage, https://hereditaryhearingloss.org

HomozygosityMapper, http://www.homozygositymapper.org/

Online Mendelian Inheritance of Man (OMIM), https://www.omim.org/

PhastCons and PhyloP, http://compgen.cshl.edu/phast/

Picard, http://broadinstitute.github.io/picard/

PyMOL, http://www.pymol.org

SHIELD, https://shield.hms.harvard.edu/

Superlink Online, http://cbl-hap.cs.technion.ac.il/superlink-snp/

Swiss model, https://swissmodel.expasy.org

World Health Organization, https://www.who.int/health-topics/hearing-loss

**Supplementary Tables**

**Table 1**: List of variants identified and verified with Sanger Sequencing as not segregating.

**Table 2**: Annotation information for candidate gene variants.
